# The Epidemiological and Molecular Aspects of Influenza H5N1 Viruses at the Human-Animal Interface in Egypt

**DOI:** 10.1371/journal.pone.0017730

**Published:** 2011-03-21

**Authors:** Ghazi Kayali, Richard J. Webby, Mariette F. Ducatez, Rabeh A. El Shesheny, Ahmed M. Kandeil, Elena A. Govorkova, Ahmed Mostafa, Mohamed A. Ali

**Affiliations:** 1 Division of Virology, Department of Infectious Diseases, St. Jude Children's Research Hospital, Memphis, Tennessee, United States of America; 2 Virology Laboratory, Environmental Research Division, National Research Centre, Cairo, Egypt; University of Liverpool, United Kingdom

## Abstract

With 119 confirmed cases between March 2006 and December 2010, Egypt ranks second among countries reporting human H5N1 influenza virus infections. In 2009–2010, Egypt reported 68 new human cases and became the new epicenter for H5N1 infections. We conducted an epidemiological and molecular analysis in order to better understand the situation in Egypt. The onset of new cases peaked annually during the winter and spring months, with majority of cases reported in the Nile Delta region. Most cases were less than 18 years old (62%) and females (60%). The overall case-fatality rate was 34% and significantly increased by age. There was a significant difference between the case-fatality rates among females and males. We observed a significant drop (p = 0.004) in case fatality rate in 2009 (10%) as compared to higher rates (36%–56%) in other years. Hospitalization within 2 or 3 days after onset of symptoms significantly decreased mortality. Molecular analysis showed that variations do occur among viruses isolated from birds as well as from humans in Egypt, and these mutations were especially noted in 2009 viruses. As the epidemiological profile of Egyptian cases differs from other countries, there is an urgent need to conduct prospective studies to enhance our understanding of incidence, prevalence, and determinants of virulence of human infections with avian H5N1 influenza viruses.

## Introduction

Preparedness for a possible influenza pandemic caused by highly pathogenic avian influenza (HPAI) A subtype H5N1 has become a global priority [1]. The continued occurrence of human infection with these viruses and breached host barrier have compounded pandemic concern. Long-term endemic influenza virus infections in poultry increase exposure risks to humans, and in turn, create opportunities for the emergence of human-adapted strains with pandemic potential [2], [3].

The first outbreak of human infection with the HPAI H5N1 virus was reported in 1997 in Hong Kong, where 18 people were infected, of whom 6 died [4]. After that outbreak, no new human cases were reported until 2003, when the virus was detected in a family recently returned to Hong Kong from mainland China. Since then, new human cases have occurred in numerous countries around the world [4]. In 2006, the first human case outside Southeast Asia was reported in Turkey. Later, cases were reported in Iraq, Egypt, Azerbaijan, Djibouti, and Nigeria [4]. In the Middle East, clade 2.2.1 H5N1 viruses were circulating among poultry, and were also responsible for the human cases [5], [6], [7].

With 119 confirmed cases up to December 2010, the largest number of cases outside Southeast Asia, Egypt ranks second among all countries reporting human infection with H5N1, following Indonesia [4]. In Egypt, it is estimated that 105 million birds are raised in rural areas and 126 million are kept in urban areas [8]. Response to outbreaks of HPAI H5N1 in Egyptian poultry focused on increasing awareness, culling infected poultry, and vaccinating the rest. Several inactivated H5 poultry vaccines with varying efficacy levels are available in the Egyptian market. The main problems in the strategy of control and prevention of avian influenza in Egypt were the lack of mass disinfection of the infected foci and failure to successfully vaccinate all poultry.

In this paper, we present the epidemiology of the laboratory-confirmed cases of H5N1 infection in Egypt. Sequence analysis of the human and avian H5N1 influenza viruses isolated between 2006 and 2010 as well as analysis of the sequences available in GenBank were conducted to understand the evolution of the Egyptian H5N1 viruses and to predict the possibility of pandemic emergence.

## Methods

### Epidemiological Analysis

Data on the laboratory-confirmed H5N1 cases in Egypt were compiled from various reports of the Epidemic and Pandemic Alert and Response Program of the World Health Organization (WHO) [9]. These reports were not intended for research and hence were inconsistent in reporting certain data variables. The available data variables included age, sex, exposure to poultry, governorate, and dates of symptoms' onset, hospitalization, and death. Where the date of onset of symptoms was not accurately provided, the month during which the case was reported was used to estimate the date of onset. All cases were confirmed by reverse transcriptase polymerase chain reaction at the Egyptian Central Public Health Laboratory and some were subsequently confirmed by the U.S. Naval Medical Research Unit No. 3 (NAMRU-3) in Cairo, Egypt [9], [10]. Since the data used was publicly available on the internet and no patient identifiers were used, approval from an ethics committee and patient consent were not sought.

The chi-square and Fisher exact tests were used to compare categorical data. Logistic and linear regressions were used to study the effect of age and sex on hospitalization and death. All statistical analysis was performed using PASW (SPSS) 18.0 software.

### Sequence and phylogenetic analysis

Avian samples were collected for diagnostic purposes by veterinarians from farms they provide care for through the period of 2006 to 2010. As those specimens were collected as part of the routine animal care, no animal care and use committee approval was sought. Five isolated viruses were included in this analysis (Table 1). All samples were tested for the presence of influenza A viruses by detection of M gene using one-step RT-PCR kit (Qiagen, Valencia, CA). Samples were subtyped using specific primers (Table 2). Comparative analysis of HA genes for all H5N1 viruses was carried out and compared with the available sequences using the NCBI influenza virus resource for both avian and human H5N1 viruses. Sequences were analyzed using the BioEdit program [11]. Sequences were aligned with ClustalW [12]. Phylogenetic analyses were carried out using MEGA version 4.0.2, with the neighbor-joining method, Poisson correction [13]. Bootstrap values (1000 replications) are indicated on the tree.

**Table 1:** Avian influenza H5N1 viruses isolated in Egypt between 2006 and 2010.

H5N1 Virus	Accession no.
A/chicken/Qalubia/1/2006	FR687275, FR687287, FR687276, FJ472343, FR687264, FR687263, FR687288, FR687299
A/turkey/Egypt/7/2007	CY055188–CY055195
A/chicken/Egypt/1/2008	FR687274, FR687286, FR687277, CY061552, FR687265, CY061553, FR687289, FR687298
A/chicken/Egypt/1/2009	FR687273, FR687285, FR687278, FR687255, FR687266, FR687262, FR687290, FR687297
A/chicken/Egypt/Q1011/2010	FR687272, FR687284, FR687279, FR687256, FR687267, FR687261, FR687291, FR687296
A/chicken/Egypt/Q1182/2010	FR687270, FR687282, FR687281, FR687258, FR687269, FR687259, FR687292, FR687295
A/chicken/Egypt/Q1185/2010	FR687271, FR687283, FR687280, FR687257, FR687268, FR687260, FR687293, FR687294

**Table 2:** Oligonucleotide primers for detection and subtyping of avian H5N1 influenza viruses.

Target gene	Primer sequences	Expected product size	Reference
M	M30F2/08: 5′-ATGAGYCTTYTAACCGAGGTCGAAACG-3′	244 bp	[40]
	M264R3/08: 5′-TGGACAAANCGTCTACGCTGCAG-3′		
HA	H5-kha-1: 5′-CCTCCAGARTATGCMTAYAAAATTGTC-3′	300–320 bp	[41]
	H5-kha-3: 5′-TACCAACCGTCTACCATKCCYTG-3′		
NA	N1-F: 5′-TTGCTTGGTCGGCAAGTGC-3′	615 bp	[42]
	N1-R: 5′-CCAGTCCACCCATTTGGATCC-3′		

### Antiviral resistance

The antiviral susceptibilities of H5N1 influenza viruses were determined by fluorescence-based NA enzyme inhibition assay using 2′-(4-methylumbelliferyl)-α-D-N-acetylneuraminic acid (MUNANA; Sigma, St. Louis, MO) at a final concentration of 100 µM as a substrate. The IC_50_ was defined as the concentration of NA inhibitor necessary to reduce NA activity by 50% relative to that of a reaction mixture containing virus but no inhibitor. Full-length NA and matrix (M2) proteins were sequenced to identify molecular markers of resistance.

## Results

### Epidemiology of human H5N1 cases

**Figure 1:**
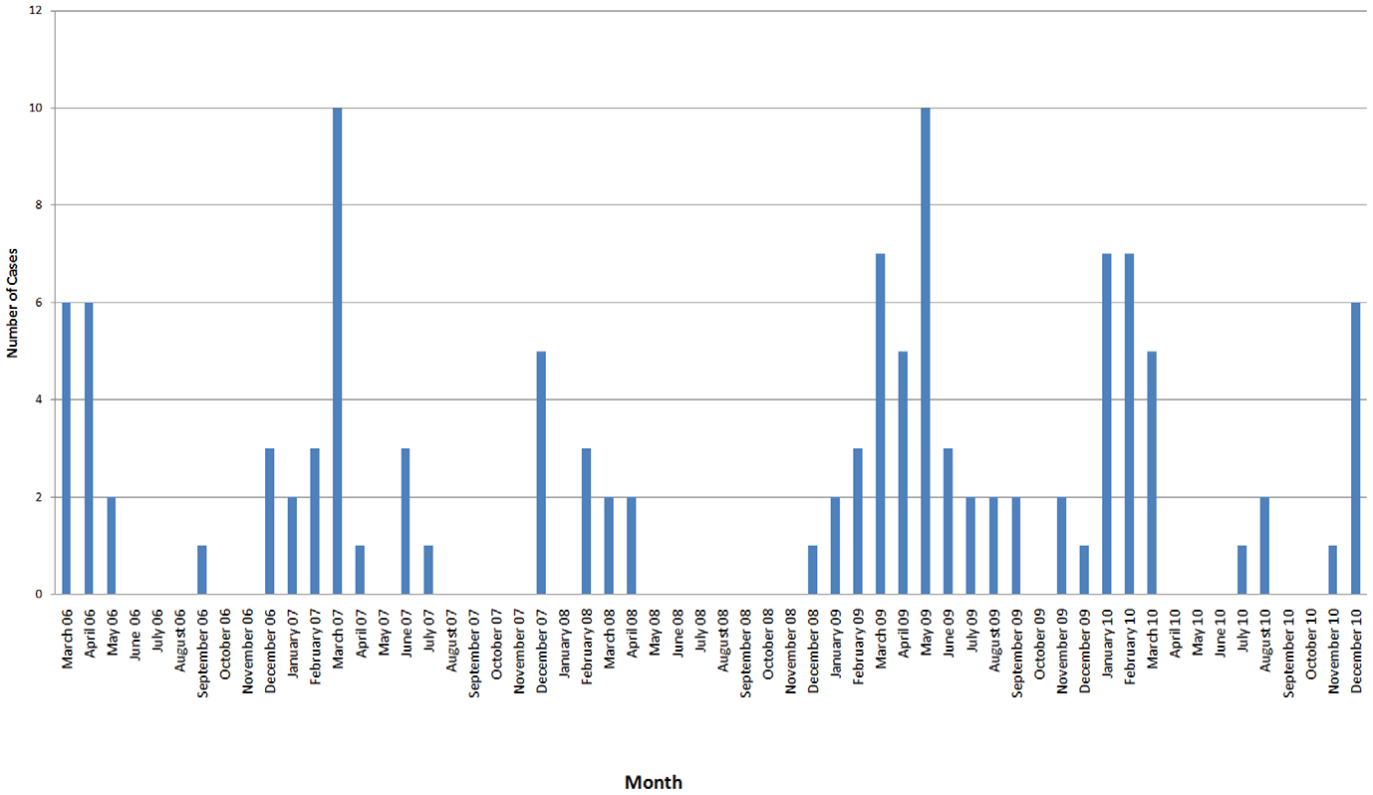
Number of human H5N1 influenza infections in Egypt by Month of Onset.

From March 2006 to December 2010, 119 human cases of infection with H5N1 were reported in Egypt. The onset of new cases peaked five times. The first was during the spring of 2006, and then during the winter and spring months of 2007, 2008, 2009, and 2010 (Figure 1). Ten cases were reported in March 2007 and May 2009, the highest number of cases in any month during the study period. The occurrence of cases in the summer months was rare, with only 17 cases reported in summer 2006 (1 case), summer 2007 (4 cases), summer 2009 (9 cases), and summer 2010 (3 cases). Three family clusters were reported: the sixth and seventh reported cases were sisters; cases 16, 17, and 18 were of the same extended family; and cases 28 and 30 were siblings.

**Figure 2:**
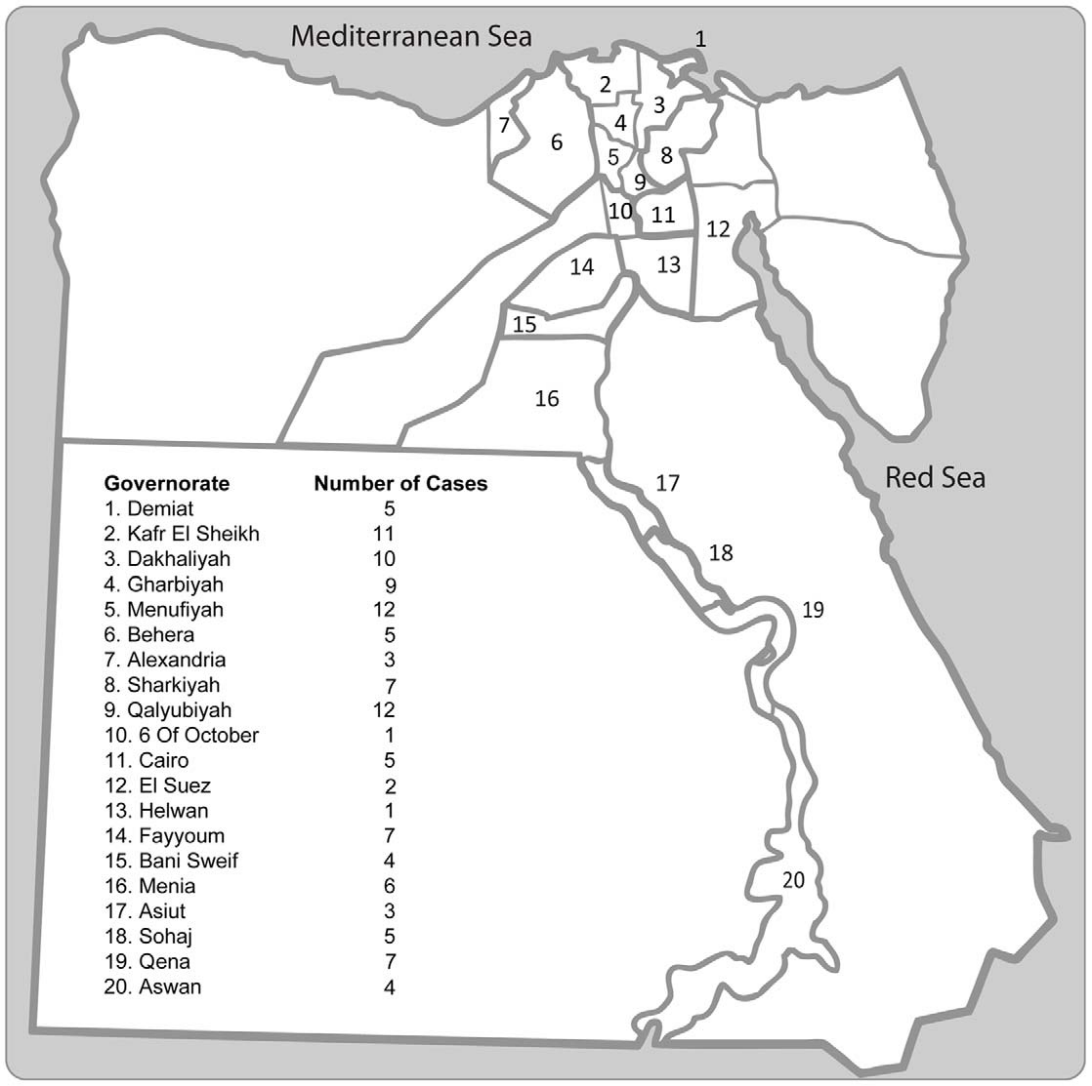
Number of human H5N1 influenza virus infections in Egypt by Governorate.

Most cases (n = 94, 79%) occurred in the northern part of the country and were concentrated in the Nile Delta region. The rest of the cases occurred over the southern governorates along the Nile, where agriculture is practiced. The northern governorates of Menufiyah and Qalyubiyah had 12 cases, the highest number of cases per governorate, followed by the Kafr El Sheikh (11 cases). In the south, the governorate of Qena had the highest number of cases (n = 7). Five cases were reported in the heavily urbanized Cairo governorate (Figure 2).

**Figure 3:**
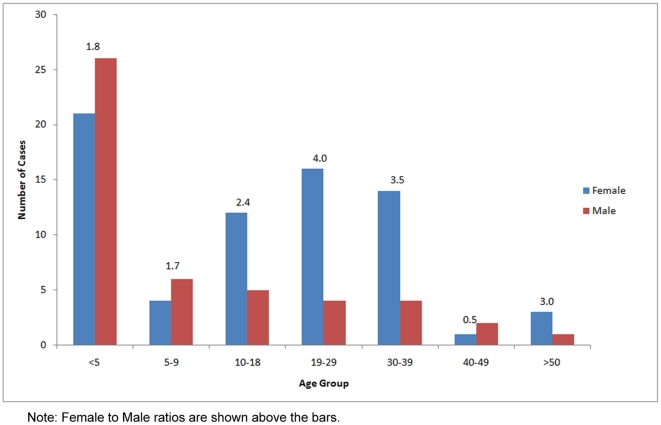
Number of human H5N1 influenza virus infections in Egypt by age and sex.

The age and sex distribution of the cases is presented in Figure 3. The mean age of these cases was 15.7 years, while the median age was 10.0 years. The youngest patient was 12 months old while the oldest was 75 years old. Most of the cases in Egypt occurred among patients less than 18 years old (62%). Children under 5 years old constituted 40% of all cases. Young adults between 19 and 39 years old accounted for 32% of all cases. Three cases were in the 40–49 age group, and 4 cases were older than 50 years.

Overall, there were more female (60%) than male cases. There were more males among cases younger than 10 years, and more females among older cases. The overall female-to-male sex ratio was 1.5. The largest sex ratio was recorded for the 19–29 years age category, in which there were 4.0 female cases for every male case. This ratio dropped to 3.5 for the 30–39 age group. For cases between 10 and 18 years, the sex ratio was 2.4. Among cases older than 40 years old, there were 1.3 female cases for every male case. There were 0.8 female case to each male case among cases less than 10 years old.

Of the 119 confirmed cases, 40 died, putting the overall case-fatality rate at 34%. Table 3 shows the case-fatality rates by age, sex, and days of hospitalization. Case-fatality rates significantly increased by age (p<0.001). Two patients younger than 5 years old and another aged between 5 and 9 years old died (4% and 10% respectively). The case-fatality rate for children between 10 and 18 years old was 53%, while 61% of adults between 19 and 49 years died. Most cases older than 50 died. There was a statistically significant difference between the case-fatality rates among females and males (p<0.001). Seven of the 48 male cases died (15%), while 33 of the 71 female cases died (47%).

**Table 3:** Case-fatality among human H5N1 influenza cases in Egypt by age group, sex, year, and the number of days between onset of symptoms and hospitalization.

		Died	Survived	Total	Case-fatality rate (%)
Age group*	<5	2	45	47	4
	5–9	1	9	10	10
	10–18	9	8	17	53
	19–29	13	7	20	65
	30–39	11	7	18	61
	40–49	1	2	3	33
	≥50	3	1	4	75
Sex†	Female	33	38	71	47
	Male	7	41	48	15
Year†	2006	10	8	18	56
	2007	9	16	25	36
	2008	4	4	8	50
	2009	4	35	39	10
	2010	13	16	29	45
Hospitalized within 2 days of onset of symptoms†	Yes	4	45	49	8
	No	22	19	41	54
Hospitalized within 3 days of onset of symptoms†	Yes	7	56	63	11
	No	19	8	27	70

*p<0.001, Fisher's exact test.

† p≤0.005, Pearson's chi-square test.

Early hospitalization had a positive effect on decreasing mortality among the cases. Due to missing data on dates of symptoms' onset and hospitalization, only 90 cases were included in the analysis of the effect of the time between the onset of symptoms and hospitalization on case-fatality. The mean time between the onset of symptoms and hospitalization was 2.7 days, ranging between hospitalization on the day of onset to 9 days later. Results from linear regression showed that age had a significant effect on hospitalization where younger cases tended to be hospitalized more rapidly independent of sex (p = 0.010). The case-fatality rate among cases who were hospitalized within 2 days of the onset of symptoms was 8%, compared with 54% among cases who were hospitalized later (p<0.001). Similarly, the case-fatality rate among cases hospitalized within 3 days was 11%, whereas 70% of cases hospitalized after 3 days of onset of symptoms died (p<0.001). Logistic regression showed that the odds ratio (adjusted for sex and age) of death among cases hospitalized after 2 days of onset was 19 (95% CI 4.0–86.3) as compared to cases who were hospitalized within 2 days. Similarly, the adjusted odds ratio of death among cases hospitalized after 3 days of onset was 19 (95% CI 5.0–74.2) as compared to cases who were hospitalized within 3 days. We observed a significant drop (p = 0.003) in case fatality rate in 2009 (10%) as compared to higher rates (36%–56%) in other years. Most of the cases in 2009 (72%) were children less than 5 years old. Logistic regression showed that mortality in 2009 remained significantly lower than other years after controlling for age (odds ratio = 0.65, 95% CI 0.4–0.98). The same effect was noticed when mortality in the years 2009 and 2010 was compared to that of the previous years (odds ratio = 0.7, 95% CI 0.5–0.97).

The pattern of avian H5N1 infection in Egypt differed slightly between humans and birds. Epidemiologically, field observations made by our and other teams of Egyptian veterinarians suggested that there was a reduction in the disease caused by H5N1 viruses in poultry between 2006 and 2010. In 2006, H5N1 virus caused severe illness in poultry that resulted in the loss of more than 90% of the flocks in Egypt. The evolution of symptoms is summarized in table 4.

**Table 4:** Symptoms of influenza H5N1 virus infection in poultry, 2006–2010.

	2006	2007	2008–2009	2010
**Mortality**	High mortality, up to100%	High mortality, up to100%	Low mortality, 30–40%	Mortality ranged between 20 to 60%
**Course of illness**	Rapid, 3–5 days	7–10 days	2–3 weeks	20–30 days
**Specific symptoms**	Redness and swelling in comb and wattles	Redness and swelling in comb and wattles	No redness or swelling in comb or wattles, but nervous symptoms appeared at the end of course	Redness, swelling in comb and wattles, and nervous symptoms at the end of course
**Common affected breeds**	Affected all types of chicken (broilers and layers)	Affected layers mainly	Affected layers mainly	Affected all types of chickens (broilers, and layers)
**Egg production**	Severe drop in egg production	Not clear due to loss of most poultry in farms	Severe drop in egg production. Egg peritonitis was the main lesion in post mortem examination	Severe drop in egg production. Egg peritonitis was the main lesion in post mortem examination

### Sequence and phylogenetic analysis

The Egyptian HPAI H5N1 virus sequences were analyzed in order to better understand whether field observations on virulence could be correlated to changes in molecular signatures known to influence viral pathogenicitiy. All the amino acid sequences of the Egyptian strains (both from the present study and publically available in GenBank as of June 1st 2010) from 2006 to 2010 had a lysine at position 627 in PB2, a marker for enhanced virulence of influenza viruses in mammals [14], except for A/chicken/Egypt/Q1182/2010 which had a glutamic acid at residue 627. Similarly, all viruses had a glutamic acid at position 92 in NS1, which has also been shown to increase viral virulence [15]. The NS1 C terminal PDZ domain ESKV was conserved for all Egyptian strains including A/chicken/Egypt/Q1182/2010, although this strain had a longer NS1 protein, the consequence of which requires further investigation [16]. Interestingly, A/chicken/Egypt/Q1185/2010 and A/chicken/ Egypt/Q1011/2010 had a 5 amino acid insertion in NS1: TMASM between residues 80 and 81 of the protein, which does not seem to match with any of the 11 influenza proteins by blast analysis. Changes in HA glycosylation sites were also investigated for the Egyptian strains but without resulting in any obvious phenotypic link. In contrast, the HA cleavage site underwent changes during this time; the dominant motif in 2006–2008 in Egypt was PQGERRRK/RKR*GLF, while 27% and 21% of the 2009 and 2010 strains, respectively, had lost a basic residue (including the A/chicken/Egypt/1/2009, A/chicken/Egypt/Q1182/2010 A/chicken/Egypt/Q1011/2010, and A/chicken/Egypt/Q1185/2010 characterized in the present study). The mutations in the cleavage site aa sequence started in some of the 2007 viruses by a change in one aa either K346R, E340K or R341K. In 2008 the mutation R341K appeared in one isolate, G339K in two isolates, and K346R in one isolate. In 2009, aa changes were more frequent in the cleavage region of the HA and more than one aa mutations were recorded in one isolate compared to the pattern of 2006 which represent the HPAIV ideal pattern. In 2010, single mutations were recorded in some isolates in addition to the presence of the HP cleavage site pattern of 2006 in all years which means that different genotypes of H5N1 virus are co-circulating in different areas.

**Figure 4:**
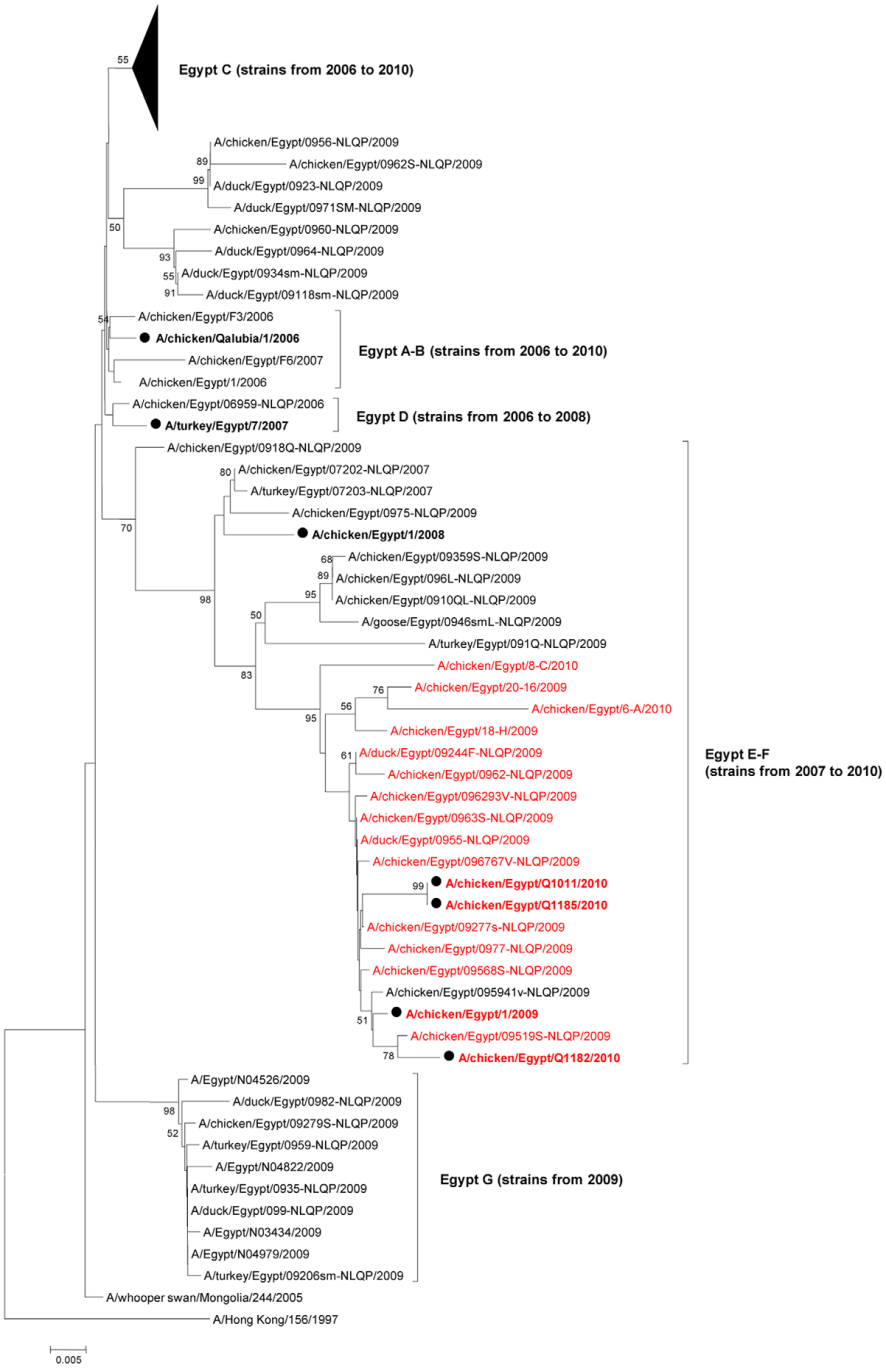
Phylogenic relationships of the HA genes of Egyptian HPAI H5N1 influenza viruses. A/chicken/Qalubia/1/2006, A/turkey/Egypt/7/2007, A/chicken/Egypt/1/2008, A/chicken/Egypt/1/2009, A/chicken/Egypt/Q1011/2010, A/chicken/Egypt/Q1182/2010, and A/chicken/Egypt/Q1185/2010 HA sequences (in bold, marked with a closed circle) were compared to all 2009–2010 HA gene sequences currently available on GenBank, as well as to a few reference 2006–2008 strains as described by Arafa et al [17]. Numbers at nodes correspond to bootstrap values >49. Strains with a basic amino acid less than their counterparts are in red font. Sublineages are indicated on the right hand side of the tree, with in parentheses for each sublineage, the year range of clustered strains.

Figure 4 highlights the evolution of Egyptian HPAI H5N1 HA. As recently described by Arafa et al., the Egyptian H5N1 strains have evolved into several sublineages within HA H5N1 clade 2.2.1 [17]. If sublineages' B and D strains seemed to have circulated from 2006 to 2008 only, sublineage A strains were still isolated in 2009, and sublineages C, E and F strains are still circulating. We believe that a new sublineage, putatively named “Egypt-G”, defined by 4 HA amino acids (H192, N309, S/N325, and R373) emerged in 2009. The following 10 strains belong to “Egypt-G”: A/Egypt/N04526/2009, A/duck/Egypt/0982-NLQP/2009, A/chicken/Egypt/09279-NLQP/2009, A/Egypt/N04822/2009, A/duck/ Egypt/099-NLQP/2009, A/Egypt/N04979/2009, A/Egypt/N03434/2009, A/turkey/Egypt/ 0935-NLQP/2009, A/turkey/Egypt/0959-NLQP/2009, A/turkey/Egypt/09206sm-NLQP /2009 (Figure 4). All viruses lacking a basic residue in the HA cleavage site belong to sublineages E-F (Figure 4). Avian and human H5N1 isolates did not cluster into specific lineages and no host-specific HA molecular marker could be identified.

Molecular markers associated with antiviral drug resistance were also examined among avian and human H5N1 influenza viruses isolated in Egypt. In contrast to previous years' isolates, 60% of the 2008 Egyptian strains as well as the 2009 and 2010 viruses sequenced in the present study (no other 2009–2010 full M2 protein sequences were available for Egyptian strains in GenBank) had the S31N mutation in M2 protein known to confer resistance to adamantanes [18], [19]. A/Egypt/14725-NAMRU3/2006, A/Egypt/ 14724-NAMRU3/2006, A/Egypt/N11981/2009, and A/turkey/Egypt/7/2007 had N294S mutation in the neuraminidase (NA), known to confer resistance to oseltamivir [20], [21]. Resistance to oseltamivir was confirmed in the NA enzyme inhibition assay *in vitro* for A/turkey/Egypt/7/2007 with a mean IC_50_ ± SD of 198.8±21.4 nM, (in contrast, A/chicken/Egypt/1/2006 had an IC_50_ of 5.8±0.2 nM).

## Discussion

The incidence of new human cases of H5N1 influenza virus infection in Egypt varied by season. Egypt has a desert climate with dry hot summers and moderate winters. Most cases occurred in the winter months, and very few were reported in the summer. This finding is in line with other reports in which incidence peaked in the winter months in the Northern Hemisphere [10]. Although such information is helpful when trying to anticipate when a surge of cases will occur, more information is needed on how climatic and other environmental factors affect disease incidence. For instance, the cold winter weather in Turkey prompts the farmers to take their poultry indoors to protect them from the cold. This increases the exposure of the household residents to the birds' secretions and feathers [22], [23]. Although the winter weather is milder in Egypt than in Turkey, local environmental factors may affect farming practices and thus are worth studying. Other environment-related practices, such as swimming in canals or ponds that poultry use, should be properly studied to understand their association with transmission of avian influenza viruses to humans [24].

Most of the cases in our study (60%) were children under 18 years old. Children under 5 years old constituted 40% of the total number of cases. In an epidemiologic analysis of WHO-confirmed human H5N1 infection cases that included the first 12 Egyptian cases, the median age was 20 years [10]. Cases in Egypt were significantly younger, with a median age of 10 years. Cases from other countries such as Vietnam, Turkey, Thailand, and Indonesia were also mostly children [22], [23], [25], [26], [27]. The occurrence of more cases among younger people could be a reflection of the overall age distribution of the populations in those countries [10]. It could also be attributed to poor hygiene and the children's behavior that bring them in direct contact with the poultry. Hygiene, behavior, and other socioeconomic factors, such as child labor and school attendance, should be studied.

Females constituted almost 60% of all cases in Egypt. This differs significantly from other studies that found that there were either approximately similar numbers of male and female cases [10] or more male cases [22], [23], [25], [26], [27]. Females in Egypt typically play the main role in tending to backyard poultry flocks. However, such an observation needs to be corroborated through detailed study of local socioeconomic variables, cultural practices, beliefs, and their association with exposure to avian influenza viruses. Understanding the role of such variables can also help fine-tune and guide preparedness efforts and public-health interventions at local levels.

The overall case-fatality rate among Egyptian cases was 34%. This is lower than the 60% case-fatality rate among WHO-confirmed cases from other countries as of January 20, 2011. Case-fatality in Egypt differed by sex and age, as more females and older cases were more likely to die. These reported case-fatality rates of avian influenza H5N1 infections in humans may be overestimated, because the extent of subclinical infections and undiagnosed cases is not really known [28]. Determining the true death rates among H5N1 infection cases requires conducting prospective, large-scale epidemiologic studies in well-defined populations.

Early hospitalization had a positive effect on survival of the cases. Similar findings have been reported by other researchers [25], [29], [30], [31]. Young age of patients was significantly associated with faster admission to the hospital after onset of the symptoms. It is possible that younger patients had more severe illness than older patients thus leading to rapid hospitalization. Alternatively, if the severity of illness is not different, adults may be delaying seeking medical care especially if the symptoms are mild. However, we were not able to ascertain the reason of delayed hospitalization among adults using available data. Delayed hospitalization, independent of sex and age, had a significant effect on death. Arabi et. al. [32] reported that H5N1 patients are usually critically ill upon admission to a hospital or shortly thereafter. Furthermore, patients infected with H5N1 virus present with a wide range of symptoms common to many other widespread illnesses [22], [24], [26], [27], [33], [34], [35]. This complicates an early differential diagnosis, and it is sometimes not until the disease is in advanced stages that such a diagnosis is made. To ensure early diagnosis and subsequently early hospitalization and treatment, primary care facilities receiving human cases of H5N1 should always suspect avian influenza as the cause of disease, especially among cases exposed to poultry.

This is not the first report on human cases of HPAI in Egypt [36], [37], [38], yet our manuscript is the first to attempt to explain the epidemiology of human cases by comparing it to molecular level changes in circulating viruses. We have also added the new cases that were reported in Egypt and were not analyzed before. Furthermore, we used regression analysis to control for age and sex when analyzing factors related to fatality and time of hospitalization. Our analysis was limited to laboratory-confirmed cases reported to the WHO. Thus, selection bias may have affected our results if some cases died or recovered before diagnosis or if false-positive or false-negative laboratory findings were included. The occurrence of cases with subclinical illness is not well studied, and this could have also affected our results. Due to lack of accurate data for some variables and absence of data for many other variables of interest, we were not able to conduct an in-depth epidemiologic investigation. Data on human infection with avian influenza reported to the WHO should be more accurate, includes linkage to sequence data, and be made more readily available for investigators; countries in which cases occur need to be more proactive at investigating cases properly [39].

The clinical symptoms among human cases reported in 2006 were similar to those in 2010, yet there was no human surveillance conducted in Egypt to ascertain the presence or absence of asymptomatic cases. The frequency of human H5N1 influenza infections parallels the pattern in poultry. Milder disease among poultry leads to mild human infection. For instance, a decrease in human case-fatality rate in 2009 was accompanied by an observed decrease in mortality among poultry. Since no host-adaptation mutation was observed so far, suggesting that the main transmission route of H5N1 in Egypt is still contact with infected birds, just as observed in the rest of the world.

We were not able to confirm that lower pathogenicity of the viruses is the main reason for increase or decrease in the the mortality rate among humans in Egypt. Considerable variation among backyard poultry was indeed observed; ducks and geese seemed significantly less affected by the virus than in 2006–2007. This is supported by the fact that mortality rates among humans in 2009 and 2010 was lower than that in the previous years after controlling for age. We were able to link (by age and date) the epidemiological and sequence data of the cases infected with A/Egypt/N04526/2009, A/Egypt/N04822/2009, A/Egypt/N04979/2009, and A/Egypt/N03434/2009 (viruses belonging to the proposed sublineage G) and found that three of these cases survived. As the virus appeared in its most virulent form in 2006, it is expected that any significant mutation, especially in the multibasic aa sequence, should lead to lower virulence. The increase in mortality in 2010 may be explained by the fact that viruses with the same virulence markers as those isolated in 2006 continued to circulate into 2010. However, other host and environmental factors that potentially affect disease severity need to be properly studied before claiming that the decrease in mortality is solely due to viral mutations.

Among cases reported in 2009 and 2010, 68 of the 121 (56%) new human cases reported were from Egypt. As Egypt became the new epicenter for human infection with H5N1, experts are concerned about the potential of H5N1 viruses circulating in Egypt to become more adapted to human-to-human transmission. These fears arise from the fact that these new viruses are less virulent and may be causing asymptomatic infections especially among adults. Thus, there is an urgent need to conduct more epidemiologic studies in Egypt and other endemic areas to enhance our understanding of incidence, prevalence, and determinants of human infections with avian influenza. It remains unknown from where the pandemic strain may emerge. Thus, sustained viral sequence comparisons and phylogenetic analyses of current HPAIV H5N1 are necessary to recognize newly emerging influenza variants and to monitor the global spread of these viruses.

## References

[1] Chen H, Smith GJ, Li KS, Wang J, Fan XH (2006). Establishment of multiple sublineages of H5N1 influenza virus in Asia: implications for pandemic control.. Proc Natl Acad Sci U S A.

[2] Matrosovich M, Zhou N, Kawaoka Y, Webster R (1999). The surface glycoproteins of H5 influenza viruses isolated from humans, chickens, and wild aquatic birds have distinguishable properties.. J Virol.

[3] Webster RG, Wright SM, Castrucci MR, Bean WJ, Kawaoka Y (1993). Influenza–a model of an emerging virus disease.. Intervirology.

[4] WHO (2009).

[5] Al-Azemi A, Bahl J, Al-Zenki S, Al-Shayji Y, Al-Amad S (2008). Avian influenza A virus (H5N1) outbreaks, Kuwait, 2007.. Emerg Infect Dis.

[6] Aly MM, Arafa A, Hassan MK (2008). Epidemiological findings of outbreaks of disease caused by highly pathogenic H5N1 avian influenza virus in poultry in Egypt during 2006.. Avian Dis.

[7] Saad MD, Ahmed LS, Gamal-Eldein MA, Fouda MK, Khalil F (2007). Possible avian influenza (H5N1) from migratory bird, Egypt.. Emerg Infect Dis.

[8] Egyptian-Cabinet (2006). Information and Decision Support Center..

[9] WHO (2009).

[10] WHO (2006). Epidemiology of WHO-confirmed human cases of avian influenza A(H5N1) infection.. Wkly Epidemiol Rec.

[11] Hall TA (1999). BioEdit: a user-friendly biological sequence alignment editor and analysis for Windows 95/98/NT.. Nucleic Acids Symp.

[12] Chenna R, Sugawara H, Koike T, Lopez R, Gibson TJ (2003). Multiple sequence alignment with the Clustal series of programs.. Nucleic Acids Res.

[13] Kumar S, Tamura K, Jakobsen IB, Nei M (2001). MEGA2: molecular evolutionary genetics analysis software.. Bioinformatics.

[14] Hatta M, Gao P, Halfmann P, Kawaoka Y (2001). Molecular basis for high virulence of Hong Kong H5N1 influenza A viruses.. Science.

[15] Seo SH, Hoffmann E, Webster RG (2002). Lethal H5N1 influenza viruses escape host anti-viral cytokine responses.. Nat Med.

[16] Jackson D, Hossain MJ, Hickman D, Perez DR, Lamb RA (2008). A new influenza virus virulence determinant: the NS1 protein four C-terminal residues modulate pathogenicity.. Proc Natl Acad Sci U S A.

[17] Arafa A, Suarez DL, Hassan MK, Aly MM Phylogenetic analysis of hemagglutinin and neuraminidase genes of highly pathogenic avian influenza H5N1 Egyptian strains isolated from 2006 to 2008 indicates heterogeneity with multiple distinct sublineages.. Avian Dis.

[18] Cheung CL, Rayner JM, Smith GJ, Wang P, Naipospos TS (2006). Distribution of amantadine-resistant H5N1 avian influenza variants in Asia.. J Infect Dis.

[19] Hurt AC, Selleck P, Komadina N, Shaw R, Brown L (2007). Susceptibility of highly pathogenic A(H5N1) avian influenza viruses to the neuraminidase inhibitors and adamantanes.. Antiviral Res.

[20] Abed Y, Baz M, Boivin G (2006). Impact of neuraminidase mutations conferring influenza resistance to neuraminidase inhibitors in the N1 and N2 genetic backgrounds.. Antivir Ther.

[21] Yen HL, Ilyushina NA, Salomon R, Hoffmann E, Webster RG (2007). Neuraminidase inhibitor-resistant recombinant A/Vietnam/1203/04 (H5N1) influenza viruses retain their replication efficiency and pathogenicity in vitro and in vivo.. J Virol.

[22] Oner AF, Bay A, Arslan S, Akdeniz H, Sahin HA (2006). Avian influenza A (H5N1) infection in eastern Turkey in 2006.. N Engl J Med.

[23] WHO (2006). Human cases of influenza A(H5N1) infection in eastern Turkey, December 2005–January 2006.. Wkly Epidemiol Rec.

[24] de Jong MD, Bach VC, Phan TQ, Vo MH, Tran TT (2005). Fatal avian influenza A (H5N1) in a child presenting with diarrhea followed by coma.. N Engl J Med.

[25] Chotpitayasunondh T, Ungchusak K, Hanshaoworakul W, Chunsuthiwat S, Sawanpanyalert P (2005). Human disease from influenza A (H5N1), Thailand, 2004.. Emerg Infect Dis.

[26] Sedyaningsih ER, Isfandari S, Setiawaty V, Rifati L, Harun S (2007). Epidemiology of cases of H5N1 virus infection in Indonesia, July 2005–June 2006.. J Infect Dis.

[27] Tran TH, Nguyen TL, Nguyen TD, Luong TS, Pham PM (2004). Avian influenza A (H5N1) in 10 patients in Vietnam.. N Engl J Med.

[28] Li FC, Choi BC, Sly T, Pak AW (2008). Finding the real case-fatality rate of H5N1 avian influenza.. J Epidemiol Community Health.

[29] Kandun IN, Tresnaningsih E, Purba WH, Lee V, Samaan G (2008). Factors associated with case fatality of human H5N1 virus infections in Indonesia: a case series.. Lancet.

[30] Thanh TT, van Doorn HR, de Jong MD (2008). Human H5N1 influenza: current insight into pathogenesis.. Int J Biochem Cell Biol.

[31] Yu H, Gao Z, Feng Z, Shu Y, Xiang N (2008). Clinical characteristics of 26 human cases of highly pathogenic avian influenza A (H5N1) virus infection in China.. PLoS ONE.

[32] Arabi Y, Gomersall CD, Ahmed QA, Boynton BR, Memish ZA (2007). The critically ill avian influenza A (H5N1) patient.. Crit Care Med.

[33] Hui DS (2008). Review of clinical symptoms and spectrum in humans with influenza A/H5N1 infection.. Respirology.

[34] Wiwanitkit V (2005). Diarrhoea as a presentation of bird flu infection: a summary on its correlation to outcome in Thai cases.. Gut.

[35] Wong SS, Yuen KY (2006). Avian influenza virus infections in humans.. Chest.

[36] Dudley JP (2009). Age-specific infection and death rates for human A(H5N1) avian influenza in Egypt.. Euro Surveill.

[37] Fasina FO, Ifende VI, Ajibade AA Avian influenza A(H5N1) in humans: lessons from Egypt.. Euro Surveill.

[38] Kandeel A, Manoncourt S, Abd el Kareem E, Mohamed Ahmed AN, El-Refaie S Zoonotic transmission of avian influenza virus (H5N1), Egypt, 2006–2009.. Emerg Infect Dis.

[39] Nicoll A (2006). Human H5N1 infections: so many cases–why so little knowledge?. Euro Surveill.

[40] WHO (2009).

[41] Slomka MJ, Coward VJ, Banks J, Londt BZ, Brown IH (2007). Identification of sensitive and specific avian influenza polymerase chain reaction methods through blind ring trials organized in the European Union.. Avian Dis.

[42] Wright KE, Wilson GA, Novosad D, Dimock C, Tan D (1995). Typing and subtyping of influenza viruses in clinical samples by PCR.. J Clin Microbiol.

